# HMGB1 released by irradiated tumor cells promotes living tumor cell proliferation via paracrine effect

**DOI:** 10.1038/s41419-018-0626-6

**Published:** 2018-05-29

**Authors:** Sijia He, Jin Cheng, Lianhui Sun, Yiwei Wang, Chuangui Wang, Xinjian Liu, Zhengxiang Zhang, Minghui Zhao, Yuntao Luo, Ling Tian, Chuanyuan Li, Qian Huang

**Affiliations:** 1 Cancer Center, Shanghai General Hospital, Shanghai Jiao Tong University School of Medicine, Shanghai, 201620 China; 2 Shanghai Key Laboratory for Pancreatic Diseases, Shanghai General Hospital, Shanghai Jiao Tong University School of Medicine, Shanghai, 201620 China; 3 Institute of Translational Medicine, Shanghai General Hospital, Shanghai Jiao Tong University School of Medicine, Shanghai, 201620 China; 4 The Department of Dermatology, Duke University Medical Center, Durham, NC 27710 USA

## Abstract

Tumor repopulation during therapy is an important cause of treatment failure. Strategies to overcome repopulation are arising in parallel with advances in the comprehension of underlying biological mechanisms. Here, we reveal a new mechanism by which high mobility group box 1 (HMGB1) released by dying cells during radiotherapy or chemotherapy could stimulate living tumor cell proliferationInhibition or genetic ablation of HMGB1 suppressed tumor cell proliferation. This effect was due to binding of HMGB1with the member receptor for advanced glycation end-products (RAGE), which activated downstream ERK and p38 signaling pathway and promoted cell proliferation. Furthermore, higher HMGB1 expression in tumor tissue correlated with poor overall survival and higher HMGB1 concentration was detected in serum of patients who accepted radiotherapy. Collectively, the results from this study suggested that interaction between dead cells and surviving cells might influence the fate of tumor. HMGB1 could be a novel tumor promoter with therapeutic and prognostic relevance in cancers.

## Introduction

Radiotherapy is often given in daily fractions with an average overall time of 5–7 weeks, which are divided off to allow the recovery of normal tissues from sublethal damage during treatment interphase. Meanwhile, surviving tumor cells can rapidly repopulate the damaged tumor in a markedly accelerated pace during the intervals between irradiation, which has been recognized as an important cause of treatment failure^1^. There is substantial experimental and clinical evidence to support the conception of repopulation during fractionated radiotherapy. Szczepanski and Trott demonstrated that regrowth of a transplantable murine adenocarcinoma was faster after irradiation than growth of non-irradiated control tumors^2^. Withers and coworkers analyzed the results of nearly 500 patients with oropharyngeal cancer and found rapid tumor regrowth during prolongation of treatment^3^. Research over the past decade has been taken to understand the molecular mechanisms of tumor repopulation after cytotoxic therapy. One of our previous studies demonstrated that dying tumor cells could stimulate the repopulation of tumors undergoing radiotherapy by activating caspase-3^4^. Caspase-3 was a cysteine protease involved at the end stage of cellular apoptotic cascade, however, in this process it activated downstream effector cytosolic calcium-independent phospholipase A_2_ (iPLA_2_) and then promoted prostaglandin E_2_ (PGE_2_) production, which potently stimulated growth of surviving tumor cells. We named this apoptosis-stimulated tumor repopulation mechanism the “Phoenix Rising” pathway. As there is a massive amount of cell death and different type of dead cell during cytotoxic cancer therapy, we wonder whether necrosis was also involved in tumor repopulation and what is the mechanism of necrosis associated tumor repopulation?

Necrosis is characterized by uncontrolled cellular and nuclear swelling in response to injury, which leads to ultimately cellular rupture^5^. With cell permeability increases, diverse intracellular molecules are released. These molecules are known as damage associated molecular patterns (DAMPs). Among these DAMPs, high mobility group box 1 (HMGB1) serves as the prototype^6^. HMGB1 was first discovered as a conserved non-histone DNA-binding protein and widely expressed in mammalian cells^7^. Structurally, HMGB1 contains two homologous DNA-binding domains (termed A and B boxes) with a negatively charged C-terminal region^8^. The biological functions of HMGB1 are dominated by its expression and subcellular location. Normally, HMGB1 is mainly localized in the nucleus, which principally regulates DNA events such as DNA repair and genome stability. While outside the nucleus, it associated with cell proliferation, autophagy, inflammation and immunity^8^. Thus, we question what is the role of HMGB1 released by necrotic cells and whether it could stimulate the proliferation of surviving cells during cytotoxic therapy?

In the present study, we provided evidence that HMGB1 released from irradiated tumor cells could stimulate the proliferation of living cells. HMGB1 inhibition by small molecule or knockout by genetic manipulating impaired this proliferation. In summary, the results from this study suggested that there was interaction between dead cells and surviving cells and which might influence the fate of tumor. HMGB1 could be a novel tumor promoter with therapeutic and prognostic relevance in cancers.

## Results

### HMGB1 was released from tumor cells after irradiation

**Fig. 1:**
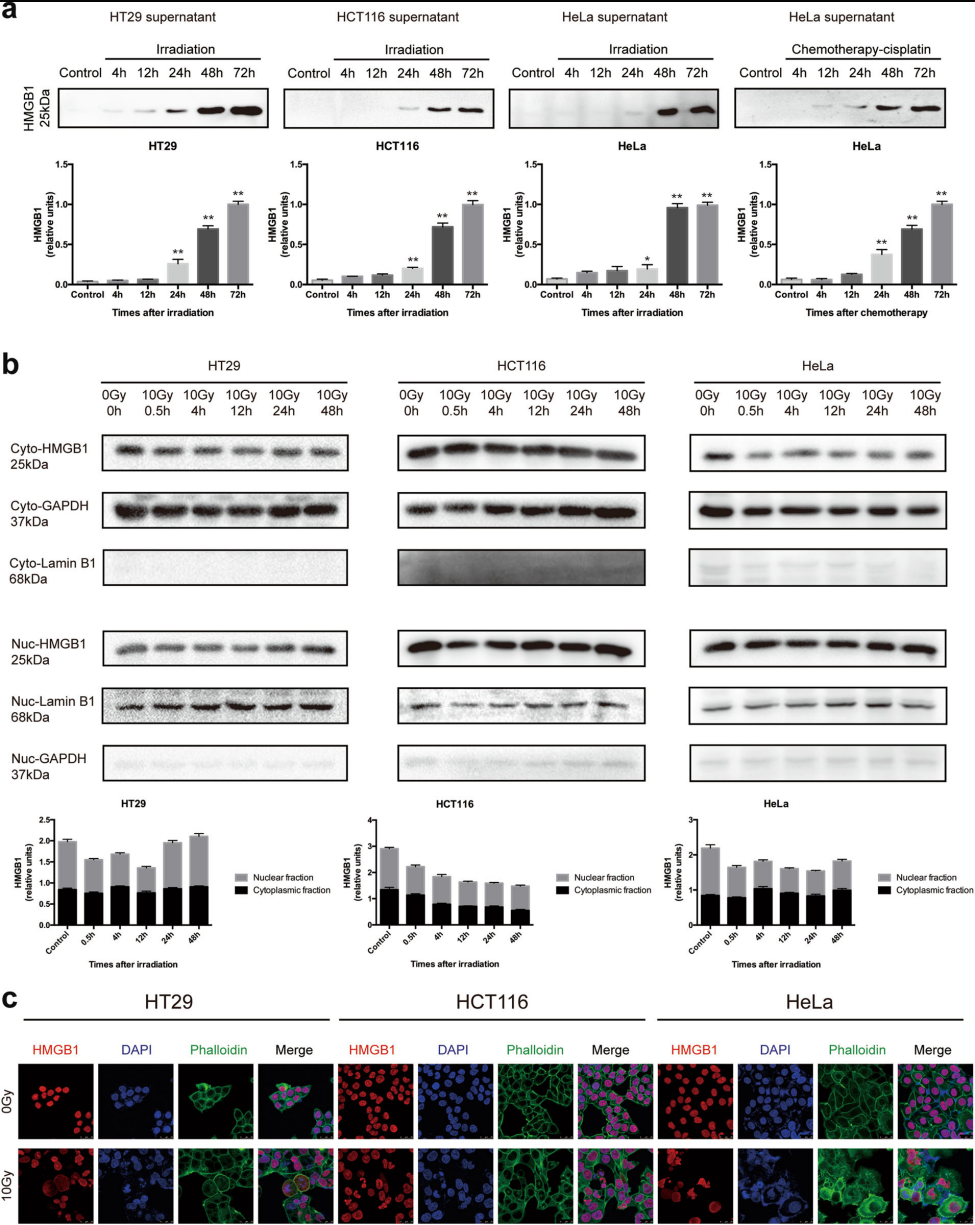
Irradiated tumor cells release HMGB1. **a** Irradiated tumor cells released HMGB1. Upper panel, the western blot results of HMGB1 in cell supernatant; Lower panel, HMGB1 relative densitometry units were calculated. **b** Cell compartmentalization. HMGB1 is localized in both nucleus and cytoplasm of tumor cells. GAPDH and Lamin B1, loading controls for the cytoplasmic and nuclear fractions, respectively. Lower panel, HMGB1 relative densitometry units were calculated. **c** Immunofluorescence staining of HMGB1. HMGB1 is detected in both nucleus and cytoplasm of irradiated tumor cells but mainly in nucleus of non-irradiation tumor cells. *p <0.05; **p <0.01

As HMGB1 is reported as a necrosis marker, we analyzed the amount of HMGB1 released in tumor cell culture medium at different time points post irradiation. At the same time, we also analyzed the expression of HMGB1 in the nucleus and cytoplasm of irradiated tumor cells at different time point after irradiation respectively. Our results showed that HMGB1 was released into the medium over time after irradiation (Fig. 1a), which was consistent with previous studies^9^. The translocation of HMGB1 from the nucleus to the cytoplasm has been reported to actively promote autocrine, which is governed by post-translational modifications such as acetylation, methylation and phosphorylation^10^. However, in this study, the expression of HMGB1 in nucleus and cytoplasm showed no significant trend post irradiation detected by western blot (Fig. 1b). Then, we analyzed the HMGB1 localization by immunofluorescence staining. The HMGB1 in non-irradiated and irradiated tumor cells was mainly localized in the nucleus, whereas in irradiated tumor cells, we found amount of multinucleate cells with altered nucleo-cytoplasmic ratio and nuclear atypic (Fig. 1c). No obvious HMGB1 translocation from nucleus to cytoplasm was seen. In order to illustrate HMGB1 releasing is a common appearance during cytotoxic therapy, we detected HMGB1 in tumor cell medium treated by chemotherapy. HMGB1 was released into the medium over time after cisplatin treatment as shown in Fig. 1a. We also did FASC analysis to see what percentage dead cells (necrosis and apoptosis) presented at different irradiation doses and the data showed that higher dose of irradiation induced more necrotic and apoptotic cells (S.1) Thus, we inferred that the primary source of HMGB1 from irradiated tumor cell culture medium was derived very likely from necrotic cells, which passively released the cellular content (including HMGB1) when cell membrane disrupted.

### Block of HMGB1 hinders tumor cell repopulation

**Fig. 2:**
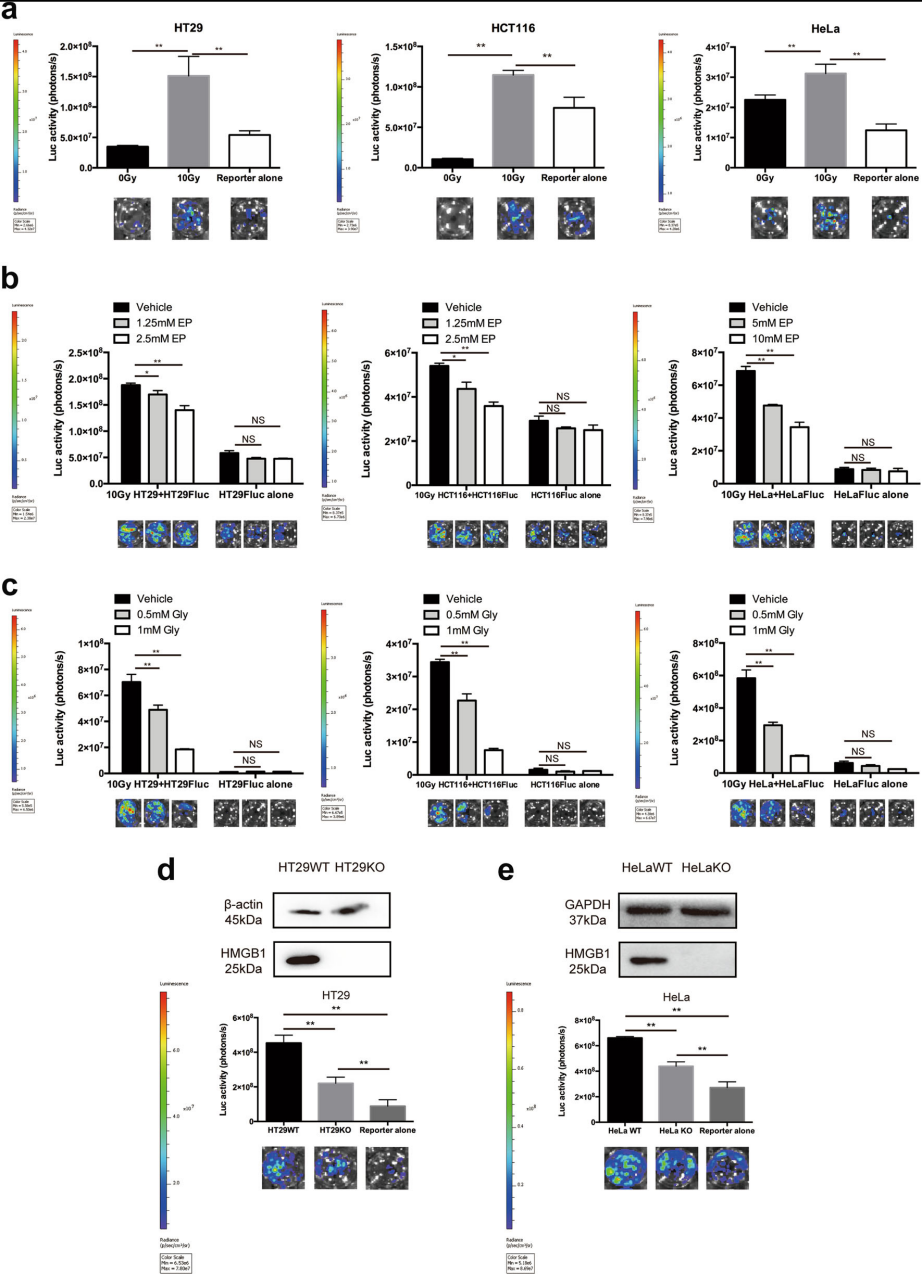
Deletion of HMGB1 hinders tumor cell repopulation. **a** Irradiated tumor cells stimulate tumor cell proliferation. Lower panel, representative bioluminescence images. **b** Inhibition of HMGB1 with ethyl pyruvate (EP) suppresses repopulation. **c** Inhibition of HMGB1 with glycyrrhizin (Gly) suppresses repopulation. **d** Knockout of HMGB1 in HT29 cells impairs repopulation. Upper panel, the HMGB1 expression of HT29 cells after deletion by CRISPR-Cas9. **e** Knockout of HMGB1 in HeLa cells impairs repopulation. Upper panel, the HMGB1 expression of HT29 cells after deletion by CRISPR-Cas9. *p <0.05; **p <0.01

Firstly, we examined whether luciferase activity and cell numbers of Fluc-labeled tumor cells had the linear correlation. As shown in Supplementary Fig. 2, the bioluminescence values of Fluc-labeled tumor cells were tightly correlated with cell numbers. We used an in vitro model which was described in our previous studies to examine tumor repopulation, in which amount of irradiated non-labeled tumor cell as “feeder” and small number of Fluc-GFP labeled living tumor cell as “reporter”. The growth of these “reporter” tumor cells was then monitored by non-invasive bioluminescence imaging. Our results showed that “feeder” cells (irradiated HT29, HCT116 and HeLa cells) promoted the proliferation of “reporter” cells (Fig. 2a), which could be suppressed by HMGB1 inhibitor (ethyl pyruvate and glycyrrhizin) (Fig. 2b, c). And higher concentration of HMGB1 inhibitors presented stronger suppression on the proliferating stimulation effect of “feeder” cells on “reporter” cells, while no toxic effect was seen in “reporter” cells alone in the same condition, suggesting that this stimulation was dose dependent on HMGB1 concentration. The importance of HMGB1 in tumor repopulation was further confirmed by knocking out the expression and function of HMGB1 by CRISPR/Cas9 technology in HT29 and HeLa cells (Fig. 2d, e). As predicted, the stimulation effect of irradiated HT29 HMGB1 KO and HeLa HMGB1 KO “feeder” cells on “reporter” cell was apparently limited (Fig. 2d, e). Our results mentioned above clearly indicated that deficiencies in HMGB1 significantly compromised the ability of irradiated tumor cells to stimulate the growth of living Fluc-labeled tumor cells.

### HMGB1 released from irradiated tumor cells promotes cancer cell repopulation via binding to RAGE

**Fig. 3:**
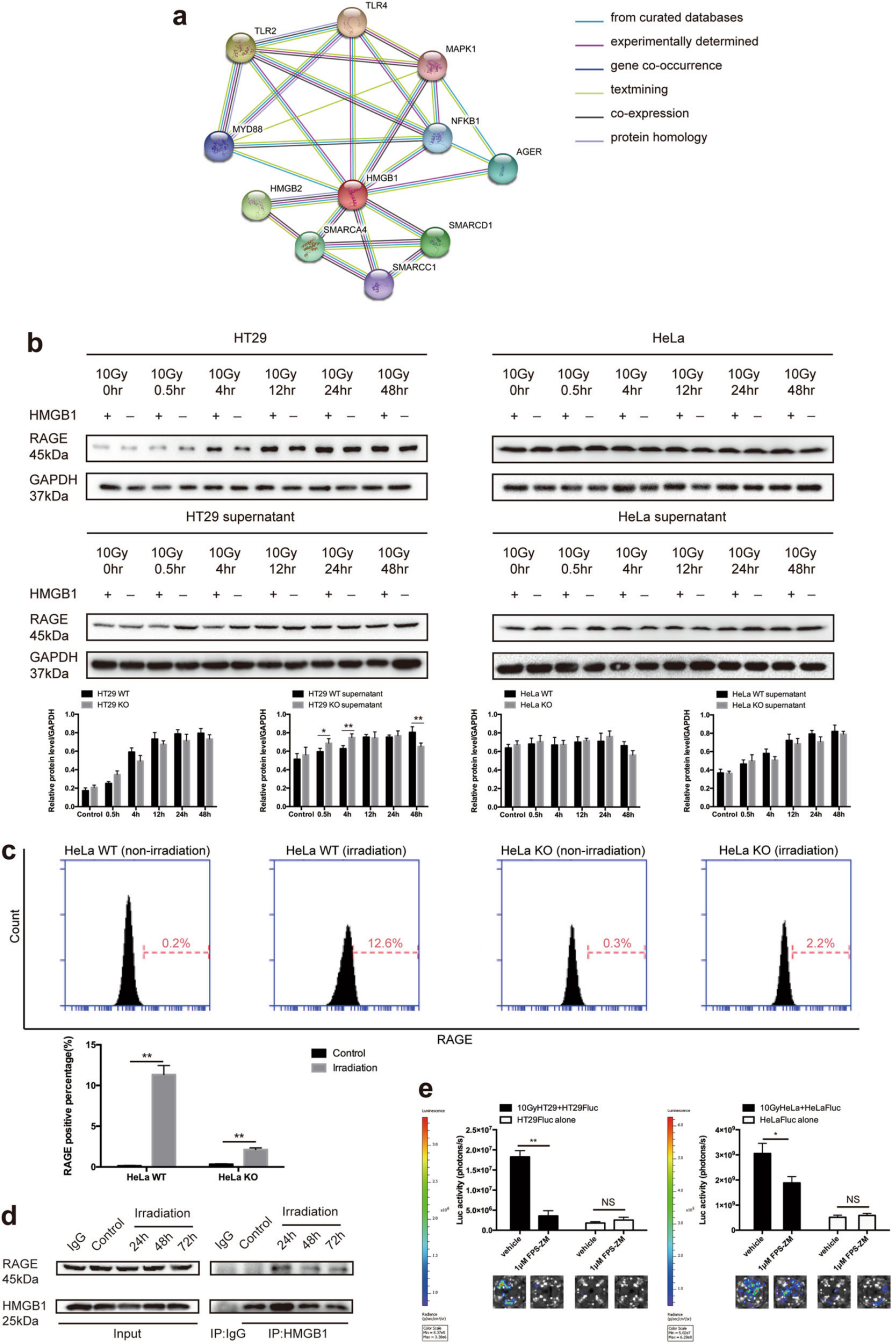
HMGB1 released from irradiated tumor cells promotes cancer cell repopulation via binding to RAGE. **a** Analysis the HMGB1 centered interaction subnetwork from STRING database. **b** Expression changes of RAGE in HT29 and HeLa cell. Left panel, expression changes of RAGE in 10Gy-irradiated HMGB1 WT and KO tumor cells at different time points. Right panel, expression changes of RAGE in HMGB1 WT tumor cells treated by 10Gy-irradiated HMGB1 WT or KO tumor cell culture supernatant collected from indicated time points. **c** Flow cytometry analysis of surface localized RAGE in HeLa cell. Lower panel, a histogram showed the percentage of RAGE positive cells. **d** Co-immunoprecipitation analysis of the binding between HMGB1 and RAGE in HeLa cells. **e** Inhibition of RAGE with FPS-ZM1 significantly recedes the ability of irradiated tumor cells to promote proliferation of Fluc-labeled tumor cells. *p <0.05; **p <0.01

To determine the cell surface binding receptors of HMGB1 released from necrotic tumor cells, we searched protein-protein association networks of HMGB1 in STRING database (https://string-db.org)^11^. Extracellular HMGB1 can bind to RAGE (also known as AGER, advanced glycosylation end product-specific receptor)^12^, TLR2 and TLR4^13^, among which RAGE is regarded as a central cell surface receptor for HMGB1^14^, which is consistent with our finding in STRING database (Fig. 3a). It is reported that RAGE expression, especially with membranous pattern, is associated with malignant potential of cancer. When RAGE is engaged by a ligand, several key cell signaling pathways can be activated, such as MAP kinases^15^ and NF-kappaB^16^, thereby reprogramming cellular properties. Impediment of the RAGE-HMGB1 interaction repressed activation of p44/p42, p38 and SAP/JNK MAP kinases, which were importantly associated with tumor proliferation^14^. Herein, we analyzed the protein expression of RAGE in irradiated HMGB1 WT and KO tumor cells, including HMGB1 WT tumor cells treated by supernatant of irradiated HMGB1 WT and KO tumor cells. Our results showed that the protein expression of RAGE increased in irradiated HT29 cell, which was more obvious in irradiated HT29 HMGB1 WT cell than irradiated HT29 HMGB1 KO cell (Fig. 3b). However, there were no significant expression change of RAGE in HT29 HMGB1 WT cell treated by supernatant of irradiated HT29 HMGB1 WT and KO cell (Fig. 3b). Moreover, RAGE expression was barely changed both in irradiated HeLa HMGB1 WT and KO cell, as well as HeLa HMGB1 WT cell treated by supernatant of irradiated HeLa HMGB1 WT or KO cell (Fig. 3b). Therefore, we hypothesized that the HMGB1-mediated stimulation effects on cell proliferation depended on the existence of RAGE rather than its protein expression level. To test this hypothesis, we first utilized flow cytometry to detect the cell surface localized RAGE on HeLa HMGB1 WT and KO cells. As a control, 0.2% and 0.3% positive rate on HeLa HMGB1 WT and KO cells were detected respectively before irradiation. Whereas, surface RAGE positive rate on HeLa HMGB1 WT cells dramatically increased to 12.6% after irradiation, but 2.2% was detected in HeLa HMGB1 KO cells (Fig. 3c). To investigate the interaction between HMGB1 and RAGE, we performed co-IP assay. As shown in Fig. 3d, RAGE and HMGB1 did not band together in non-irradiation cells, while significant association of HMGB1 and RAGE was detected after irradiation, demonstrating that RAGE was downstream receptor of HMGB1 and possibly activated after irradiation. Therefore, we used inhibitor of RAGE (FPS-ZM1) in tumor cell repopulation model and found that the repopulation was significantly suppressed (Fig. 3e), further proving that RAGE participated in HMGB1-mediated proliferating stimulation.

### Extracellular HMGB1 activates MAPK signaling pathway and promotes surviving cell proliferation

**Fig. 4:**
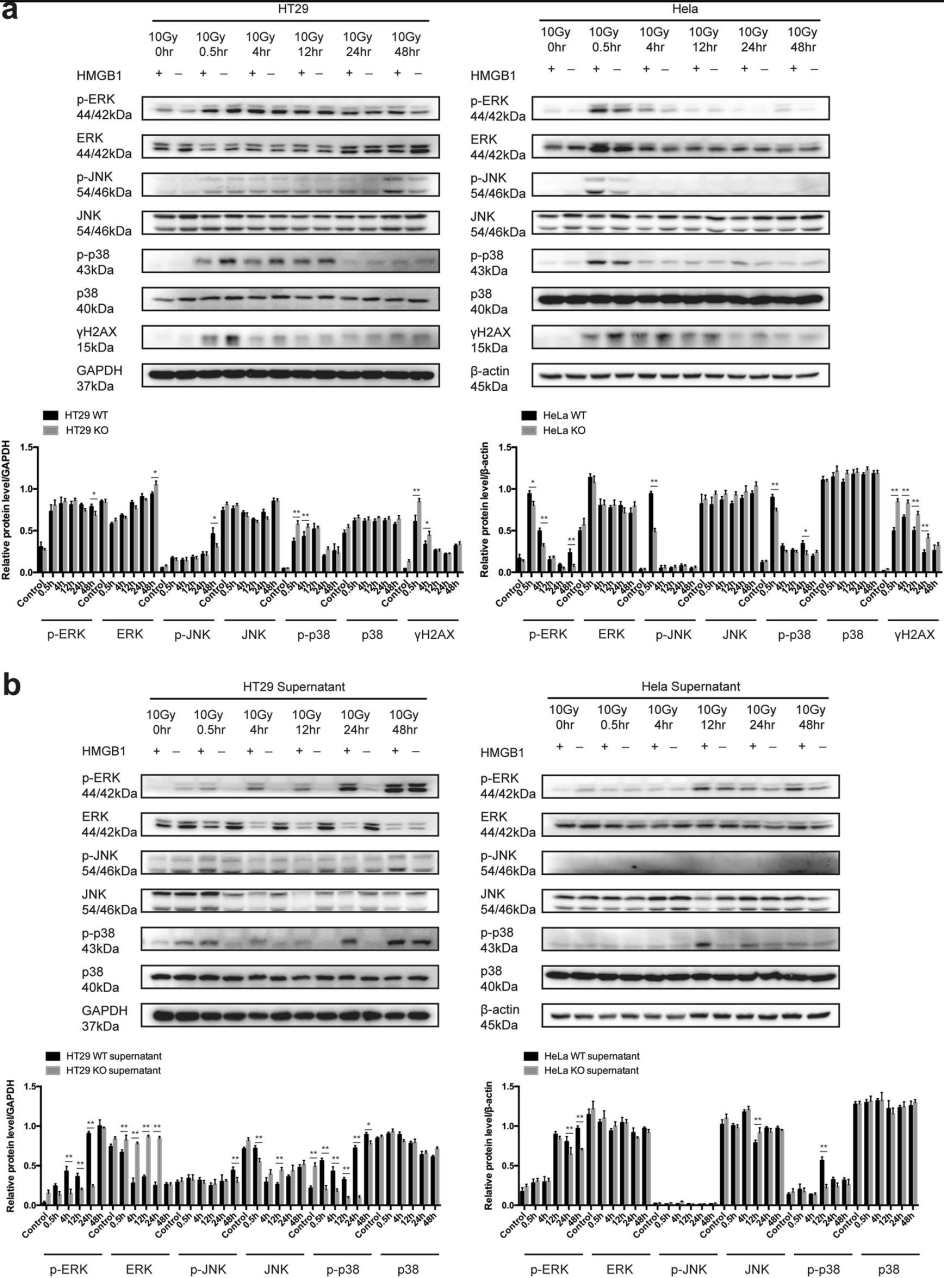
Extracellular HMGB1 activates ERK and p38 signaling pathway to promote surviving cell proliferation. **a** Left panel, expression changes of ERK, JNK, p38, and γH2AX in irradiated HT29 HMGB1 WT and KO cells. Right panel, expression changes of ERK, JNK, p38 and γ-H2AX in HeLa HMGB1 WT and KO cells. **b** Left panel, expression changes of ERK, JNK and p38 in HT29 HMGB1 WT cells treated by 10Gy-irradiated HT29 HMGB1 WT or KO cell culture supernatant collected from indicated time points. Right panel, expression changes of ERK, JNK and p38 in HeLa HMGB1 WT cells treated by 10Gy-irradiated HeLa HMGB1 WT or KO cell culture supernatant collected from indicated time points. *p <0.05; **p <0.01

To investigate the mechanistic basis for the growth stimulation effects of HMGB1 on tumor cells, we tested the contribution of ERK signaling pathway according to STRING database results (Fig. 3a), which formed major cell-proliferation signaling pathways from the cell surface to the nucleus^17^. The changes in the relative levels of protein expression of activated forms of ERK in irradiated HMGB1 WT and KO tumor cells, including HMGB1 WT tumor cells treated by supernatant derived from irradiated HMGB1 WT and KO tumor cells. As revealed by western blotting, HT29 and HeLa cells displayed elevated level of ERK signaling activation after irradiation, in which HMGB1 KO tumor cells rendered less visible (Fig. 4a, b). Similar induction was observed in HMGB1 WT tumor cells treated by irradiated HMGB1 WT or KO cell culture supernatant, although the activation timing and intensity were slightly different (Fig. 4a, b). γH2AX is a well-known DNA damage marker. In this study, irradiated tumor cells showed different levels of γH2AX protein expression, and HMGB1 KO tumor cells exerted severe DNA damage (Fig. 4a, b). Since ERK, p38 and JNK are family members of MAPK, we wondered whether p38 and JNK also underwent the similar alteration. We measured whether the activation of p38 or JNK signaling pathway in the same condition. The irradiation did not induce JNK activation in both HeLa and HT29 cells, while p38 showed short time activation.(Fig. 4a, b). In general, the downstream molecule of HMGB1/RAGE was mainly involved ERK and p38 signaling pathway, while JNK signaling pathway might not be involved.

### HMGB1 is highly expressed in tumor tissues and its expression correlates with worse clinical prognosis

**Fig. 5:**
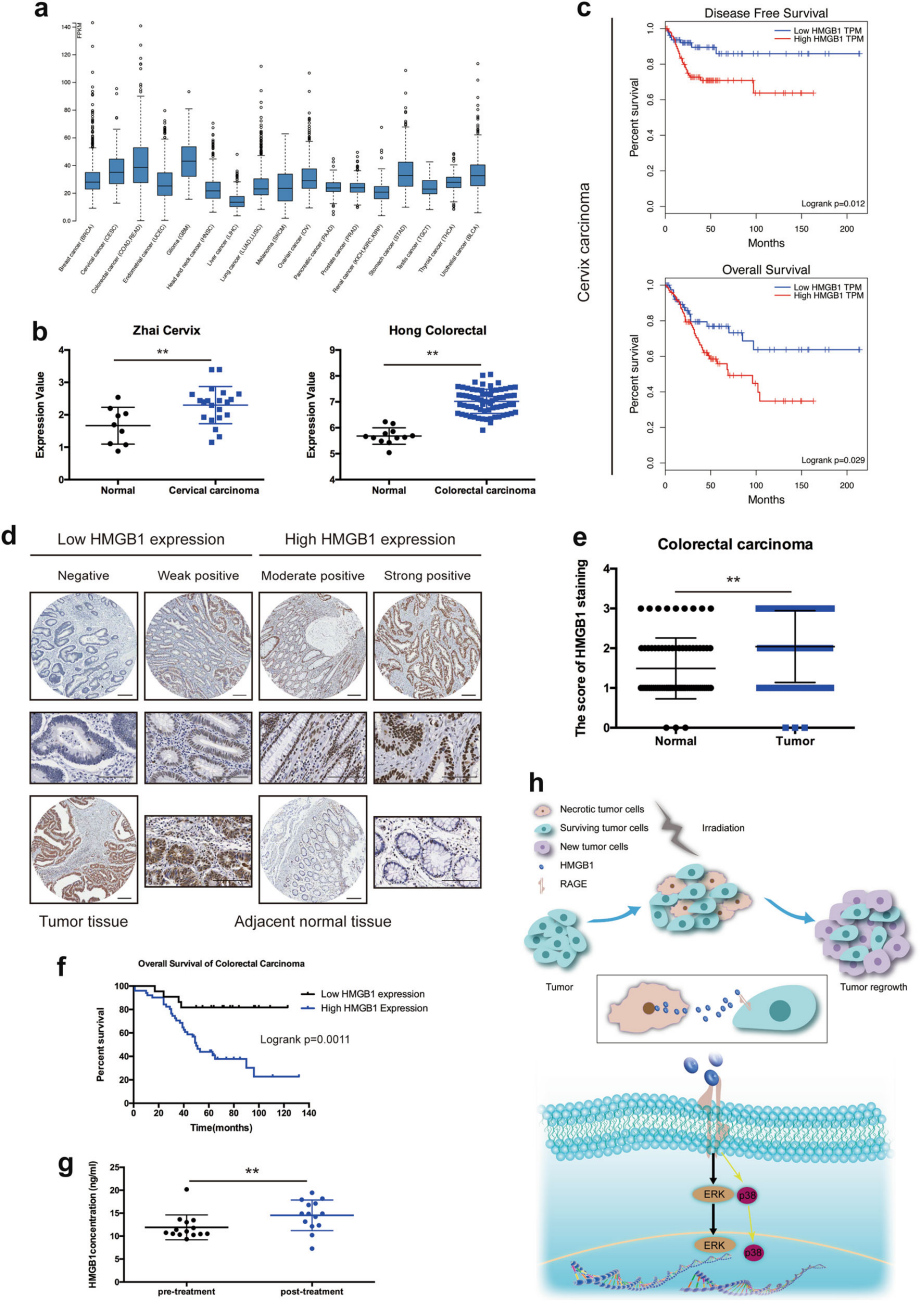
HMGB1 is highly expressed in tumor tissues and its expression correlates with worse clinical prognosis. **a** Box plots summarize the RNA expression levels of HMGB1 across 17 different cancer types. **b** The expression of HMGB1 in normal and tumor tissue (cervical carcinoma and colorectal carcinoma) from Oncomine database. **c** The survival analysis of patients with cervical carcinoma from GEPIA database. Upper panel, the overall survival of patients with cervical carcinoma. Lower panel, the disease free survival of patients with cervical carcinoma. **d** Immunohistochemical staining of HMGB1 in colorectal cancer and adjacent normal tissue. Scale bar: 200 μm (100 μm in the enlarged view). **e** The score of HMGB1 staining in colorectal cancer tissue and adjacent normal tissue. **f** The Kaplan–Meier survival analysis in 73 cases of patients with colorectal cancer. **g** HMGB1 ELISA analysis of patient’s serum pre- and post-treatment. **h** Schematic depicting the role of HMGB1 released from necrotic cells in promoting tumor proliferation through binding to RAGE and subsequently activate ERK and p38 signaling in surviving cells. *p <0.05; **p <0.01

In order to determine the relevant of our newly discovered pathway for necrotic cell-mediated tumor repopulation in human cancer, we evaluated HMGB1 status in cancer patients, especially in colorectal cancer patients. First of all, we analyzed the RNA expression of HMGB1 across 17 cancer types on HPA and found that HMGB1 was widely expressed in these cancers (Fig. 5a). Additionally, HMGB1 expression was significantly higher in colorectal (*p* < 0.01) and cervical tumors (*p* < 0.01) than normal tissues according to Oncomine database (Fig. 5b), which was consistent with our TMA results (Fig. 5d, e). All these clinical characteristics of CRC patients from TMA were shown in Table 1. To further investigate the clinical relevance of HMGB1, we analyzed HMGB1 staining in primary colon tumor samples of 73 patients from TMA and found that high HMGB1 expression was positively correlated with tumor grade (*p* = 0.042, Table 1). Subsequent Kaplan-Meier analysis of patients with cervical carcinoma from GEPIA demonstrated that patients with high HMGB1 expression levels displayed significantly poorer overall survival (*p* = 0.029) and disease free survival (*p* = 0.012) than those with low HMGB1 expression levels (Fig. 5c). As to CRC patients, Kaplan-Meier analysis from our TMA results revealed that patients with high HMGB1 expression levels also exhibited significantly poorer overall survival (*p* = 0.0011) (Fig. 5f). Multivariate analysis using Cox proportional hazards model showed that tumor grade (*p* = 0.047) and HMGB1 (*p* = 0.011) were independent prognostic factors in CRC patients (Table 2). Therefore, high HMGB1 expression can be used as a potential clinical marker, linking to aggressiveness and disease progression in patients with cancer. In addition, we detected the protein expression level of HMGB1 in the patient’s serum samples, which were collected before and after radiotherapy. Our results showed that the concentration of HMGB1 was higher in post-treatment serum samples (Fig. 5g). Tumor irradiation led to HMGB1 releasing as well, in accordance with our finding in in vitro tumor cells, indicating that our theory (HMGB1-mediated tumor repopulation) might also be true in clinic.

**Table 1:** Correlation between HMGB1 protein expression and clinicopathologic features of the patients with CRC

Variables	Cases (*n* = 73)	HMGB1 expression				*χ* ^2^	*p*-value
		−	+	++	+++		
Age						5.599	0.133
<61	36	2	12	13	9		
≥61	37	1	7	10	19		
Gender						0.415	0.937
Male	42	2	10	13	17		
Female	31	1	9	10	11		
Grade						13.063	0.042
I	24	3	7	5	9		
II	28	0	5	14	9		
III	21	0	7	4	10		
TNM stage						12.677	0.178
I	18	2	8	5	3		
II	22	0	4	8	10		
III	28	1	7	9	11		
IV	5	0	0	1	4

**Table 2:** Univariate and multivariate survival analysis of overall survival in 73 patients with CRC

Variables	Univariate analysis	Multivariate analysis		
	*p*-value	*p*-value	RR	95%CI
HMGB1 expression (low vs high)	0.003	0.011	0.230	0.074–0.710
Age (<61 vs ≥61)	0.445	0.482	0.751	0.337–1.672
Gender (male vs female)	0.886	0.445	1.336	0.635–2.809
Grade (I vs II–III)	0.018	0.047	0.396	0.158–0.988
TNM stage (I–II vs III–IV)	0.001	0.469	2.629	0.192–36.088
Lymph nodes (negative vs positive)	0.001	0.071	0.098	0.008–1.219
Metastasis (negative vs positive)	0.017	0.144	0.362	0.093–1.416

## Discussion

According to the morphologies of the dying cells, there are three types of cell death, which have been generally adopted. These are apoptotic cell death, “autophagic” cell death and necrotic cell death^18^. After cytotoxic therapy, soluble factors are released from dying cells, and then recruit immune cells for clearance^18^. Paradoxically, some of these factors are growth stimulating factors, which attracts attention on interactions between dying and living cells. Previous studies have manifested that apoptotic tumor cells stimulate a small number of surviving tumor cells repopulating which is related to caspase3/iPLA_2_ /PGE_2_ pathway^4,19^, caspase3/7-PKCδ-Akt/p38 MAPK^20^, and caspase3/PKCδ/p38/MNK1 signaling pathway^21^. Moreover, chemotherapy-induced apoptosis can release PGE_2_ to promote surrounding cancer stem cell proliferation, which contributes to residual tumor repopulation between chemotherapy cycles^22^. In addition to apoptotic cells, there are amount of necrotic cells in response to excessive damage. Importantly, necrosis also occurs if a cell dying by apoptosis is not rapid engulfed and cleared^18^. It is known that necrotic cells and their associated DAMPs are immunogenic and pro-inflammatory, however, the role of this cell death form in the repopulation remains poorly investigated.

HMGB1 is an abundant non-histone nuclear protein and considered as a central regulatory protein in cancer development^23^. As a matter of fact, HMGB1 expression is enhancive in many types of cancer, which interrelates with tumor malignancy and poor prognosis^24^. Once released, HMGB1 can interact with a plethora of cell surface receptors and exert a series of cell regulating effects from cell survival to cell death. In apoptotic cells, HMGB1 is bound firmly to chromatin owing to generalized underacetylation of histone. When chromatin deacetylation is restrained, HMGB1 can be released outside cells. Therefore, apoptotic cells are programmed to withhold the signal that is disseminated by cells under excessive stress, which make the extracellular HMGB1 a distinctive signal for necrosis. Although the direct role of HMGB1 in stimulating tumor repopulation has not been described before, many studies have been published on other roles of HMGB1 in mammalian biology. For instance, HMGB1 has been implicated in a wide range of cellular responses, such as inflammation, cell proliferation, migration and differentiation^10^. Several receptors and molecules, such as the receptor for advanced glycation end-products (RAGE), toll-like receptors, syndecan and thrombomodulin, have been proved to activate downstream signaling upon binding to HMGB1 either directly or indirectly via forming a complex with other molecules^10,23,25^.

Our studies converged on a critical role for HMGB1 in tumor cell proliferation after irradiation. We demonstrated that HMGB1 released from irradiated tumor cells promoted surviving tumor cell proliferation via HMGB1/RAGE/ERK signaling pathway (Fig. 5f). Administration of either HMGB1 inhibitors or genetic ablation of HMGB1 abolished HMGB1-mediated tumor cell proliferation. This stimulation required the binding between HMGB1 and RAGE on cell surface, which activated downstream protein ERK and p38 to promote cell proliferation. Analysis of HMGB1 expression in cancer patients demonstrated that higher expression levle of HMGB1 was detected in serum of patients who accepted radiotherapy. Furthermore, there was a positive correlation between the high expression of HMGB1 and the malignant phenotype in CRC patients. Higher expression of HMGB1 in tumor tissue predicted poor overall survival.

Overall, we discovered this newly mechanism of the interaction between dead cells and surviving cells, which might influence the fate of tumor. According to our results, radiation-based combination therapy with a molecular HMGB1 inhibitor could serve as a potential option for cancer treatment. This mechanism not only has profound implications in our understanding of cancer biology, it also provides new insights into how to improve our limited treatments for patients with cancer. It is fairly clear that when cells die they are not “forgotten”. Further investigation is needed to figure out how dying cells impact their surrounding cells.

## Materials and methods

### Cell culture and treatment

Human colon carcinoma cell lines HT29, HCT116 and human cervical carcinoma cell line HeLa cells were purchased from the American Type Culture Collection. All of these cells were cultured in Dulbecco’s modified Eagle media (DMEM) supplemented with 10% fetal bovine serum (FBS) and 1% penicillin-streptomycin. The cell lines were maintained in a 5% CO_2_-humidified atmosphere at 37 °C. Irradiation with X-ray was carried out at a dose rate of 3.6 Gy/min using a linear accelerator (Siemens, Germany). Cells were seeded in a 6-well cell culture plate and incubated for 24 h after irradiation. The cell culture supernatant from HMGB1 wide-type (WT) and knockout (KO) tumor cells for various time periods followed by irradiation was also collected for HMGB1 detection and non-irradiated HMGB1 WT tumor cell treatment.

### Gene transduction and HMGB1 knockout via CRISPR/Cas9

The pLEX lentiviral vector system (Open Biosystem, Huntsville, AL, USA) was used to incorporate foreign genes into target cells as previous described^21^. The firefly luciferase (Fluc) and green fluorescent protein (GFP) fusion gene was kindly provided by Prof. Chuanyuan Li. HT29Fluc, HCT116Fluc and HeLaFluc were constructed through lentivirus infection and subsequent puromycin selection at 2–3 μg/ml.

HMGB1 knockout cell line was generated as described^26^. In detail, 293 T cells (1 × 10^6^ cells per well; 6-well plate) were transfected with lentiviral construct expressing two gRNAs (gRNA1: 5′ GGAAGAGTCGACTCGCTT 3′, gRNA2: 5′ GTGATGTTGCGAAGAAAC 3′) and Cas9 endonuclease together with ecotropic packaging plasmids, using Lipofectamine 2000 (Invitrogen, Carlsbad, CA) according to manufacturer’s instructions. Media containing viruses were collected 48 h after transfection and then utilized to infect HeLa and HT29 cells. Infected cells were selected with puromycin (1 μg/ml) for 2 weeks and then collected for further analysis. Single colonies were picked out, subcultivated, and HMGB1 expression was tested by western blot. The cell lines with no detectable HMGB1 signal were kept for further experiments.

### Construction of repopulation model in vitro

Irradiated tumor cells with 10 Gy of X-ray (“feeder” cells) were seeded into 24-well plates (4–8 × 10^4^ cells per well). After cell attachment (overnight), the non-irradiated Fluc-labeled tumor cells (“reporter” cells) were seeded at a density of 500 cells/well. Then, feeder cells and reporter cells co-cultured for 6–8 days by replacing the cell culture medium with fresh DMEM containing 2% FBS every two days. The growth of the small number of reporter cells was monitored through non-invasive bioluminescence imaging machine IVIS Lumina Series III (PerkinElmer, USA).

### Cell lysates preparation and western blotting

The cytoplasmic and nuclear fractions separated using the protein extraction Kit (BioVision, Palo Alto, USA) according to the manufacturer’s instructions. Whole cell lysates were prepared in a buffer containing protease and phosphatase inhibitor mixture (Roche Molecular Biochemicals, Mannheim, Germany) at 4 °C. Western blotting analysis was performed as previously described^20^. Primary antibodies were used: HMGB1, RAGE, β-actin, GAPDH, Lamin B1, phospho-ERK, ERK, and γH2AX (Cell Signaling Technology, USA, except HMGB1 and RAGE from Abcam, USA).

### Immunofluorescence staining

Cells were placed on confocal dishes and incubated at 37 °C overnight. The cells were irradiated and cultured for certain time, the medium was removed and the cells were fixed with 4% formalin for immunofluorescence staining as described^4^. The primary antibody used was HMGB1 (Abcam, USA). Visualization of actin filaments was accomplished by staining the cell with phalloidine (AAT Bioquest, CA). Cell nuclei were stained with DAPI (Vector Laboratories, Burlingame, CA). Images were acquired on a confocal spinning disk microscope (Leica, Germany).

### Flow cytometric analysis

Cells were dissociated from 6 cm culture dishes with trypsin, and washed three times with cold PBS. Then the cell pellets were suspended with 200 μl Annexin V-FITC binding buffer and incubated with anti-RAGE primary antibody (1:200) at room temperature for 1 h. After that, cells were washed three times with cold PBS, incubated with secondary antibody (1:400) in the dark at room temperature for 30 min. Finally, the cells were washed again with cold PBS thrice and the RAGE expression positive rate was measured by Accuri C6 Flow cytometer (BD Biosciences, CA, USA). Each experiment was performed independently for three times.

### Co-immunoprecipitation analysis

Cells in 10 cm culture dishes were washed with cold PBS for three times, lysed in 500 μl buffer (50 mM Tris-HCl pH 8.0, 5 mM EDTA, 150 mM NaCl, 0.5% NP-40, 1 mM PMSF) for 30 min on ice, and then lysates were centrifuged for 5 min at 14,000 rpm, removed insoluble precipitate. Supernatants (60 μl) were collected as input. Cell lysates were pre-cleared with 60 μl of 50% protein A/G agarose (Santa Cruz Biotechnology, Inc.) for 30 min. Then cell lysates were centrifugated at 2000 rpm for 1 min to remove protein A/G agarose and immunoprecipitated with anti-HMGB1 antibody or IgG plus protein A/G agarose overnight at 4℃. The beads were washed with lysis buffer for three times, boiled in SDS sample buffer, and analyzed by western blotting with antibodies against HMGB1 and RAGE.

### ELISA assay

HMGB1 concentration in patient serum was quantified by a human HMGB1 ELISA kit (IBL, Hamburg, Germany) according to the manufacturer’s instructions. All samples were repeated thrice.

### Public datasets mining procedures

To investigate the regulation modes of HMGB1, we constructed HMGB1 centered interaction subnetwork from high quality STRING protein interaction database (https://string-db.org)^11^, which collects and integrates all functional interactions between the expressed proteins by uniting forecasted and known protein-protein association data.

GEPIA (Gene Expression Profiling Interactive Analysis) (http://gepia.cancer-pku.cn) is a web-based tool to implement fast and customizable functionality on the basis of TCGA and GTEx data^27^. GEPIA also performs survival analysis base on gene expression levels, which we select custom cancer types for overall and disease-free survival analysis^27^. In addition, we queried HMGB1 differential expression results of tumor and normal tissue in virtue of Oncomine database (http://www.oncomine.org.)^28^.

To explore the expression of HMGB1 in human tumors, we searched HMGB1 straightforward on the Human Protein Atlas (HPA) (https://www.proteinatlas.org)^29^. RNA expression levels across 17 cancer types are summarized as median FPKM (number Fragments Per Kilobase of exon per Million reads).

### Tissue microarray staining and analysis

An immunohistochemical staining was performed with anti-HMGB1 antibody in a tissue microarray (TMA) including 73 paired human CRC and adjacent normal samples. This TMA (ZL-COC1504) was purchased from Shanghai Superbiotek company. Images were taken by the Scanner Aperio Scanscope CS. The HMGB1 staining score of each sample was determined according to the percentage of immunopositive cells and the immunostaining intensity. Score 0 presents negative (−), 1 as weak positive (+), 2 as moderate positive (++), and 3 as strong positive (+++). Scoring with 0 and 1 represented lower HMGB1 expression, whereas 2 and 3 represented higher expression of HMGB1.

### Statistical analysis

Results are expressed as the means ± SD. Differences were analyzed by *t*-test or ANOVA. The median difference in paired data was tested by the Wilcoxon signed rank test. Survival analyses were conducted using the Kaplan–Meier method and log-rank test. Cox proportional hazards regression was utilized to model survival after adjusting for gender, age, grade and stage. The propriety of the Cox regression model was assessed using numerical methods and graphical displays. Results are considered significant at *p* *<* 0.05.

## Electronic supplementary material

Supplemental Material 1

Supplemental Material 2

Supplementary figure legends
