# Supplementary figures and images for: HMGB1 released by irradiated tumor cells promotes living tumor cell proliferation via paracrine effect
Source: Cell Death Dis. 2018 May 29;9(6):648. doi: 10.1038/s41419-018-0626-6 (PMC5974346; doi:10.1038/s41419-018-0626-6)

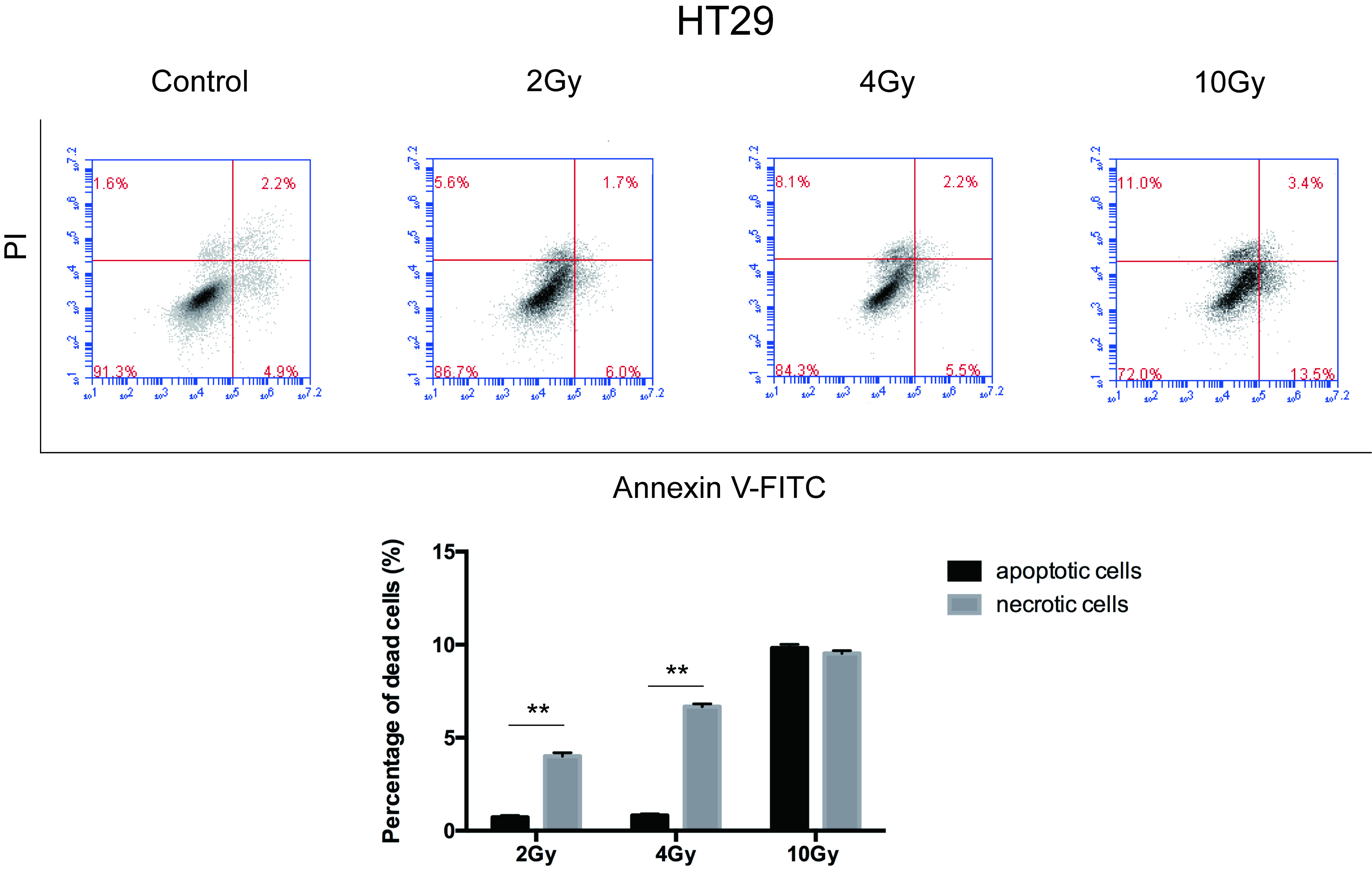

Supplement: Supplementary file 1 — Supplemental Material 1 [file 41419_2018_626_MOESM1_ESM.tif]

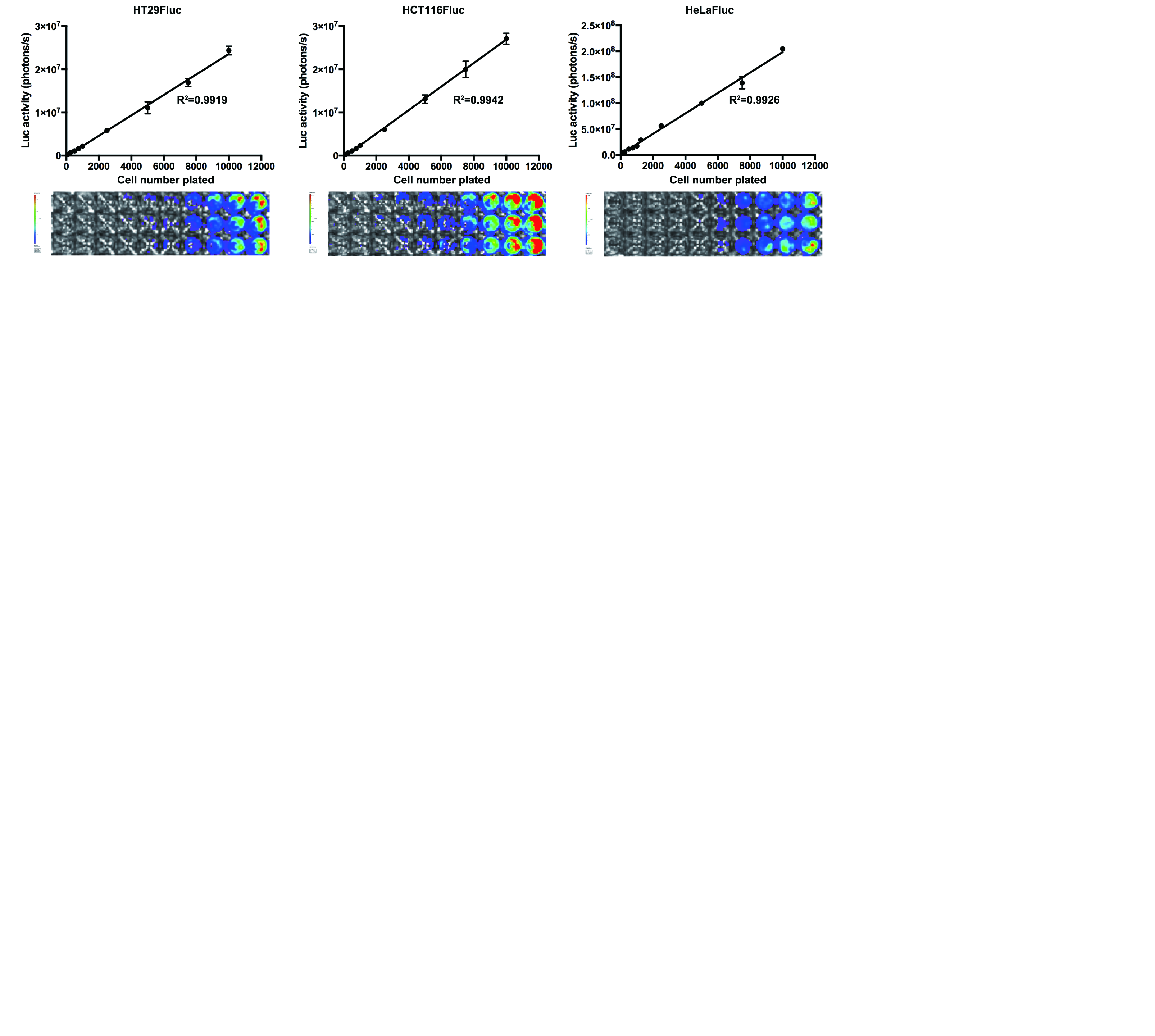

Supplement: Supplementary file 2 — Supplemental Material 2 [file 41419_2018_626_MOESM2_ESM.tif]
