# Supplementary material for: HMGB1 released by irradiated tumor cells promotes living tumor cell proliferation via paracrine effect
Source: Cell Death Dis. 2018 May 29;9(6):648. doi: 10.1038/s41419-018-0626-6 (PMC5974346; doi:10.1038/s41419-018-0626-6)
Supplement: Supplementary file 3 — Supplementary figure legends [file 41419_2018_626_MOESM3_ESM.docx]

Supplementary 1. Irradiation induced tumor cell death in a dose manner. **p*＜0.05, ***p*＜0.01.

Supplementary 2. Linear correlation between luciferase activity of Fluc-labeled tumor cells and cell number plated.
